# Weight self-stigma and engagement among obese students in a physical education class

**DOI:** 10.3389/fpsyg.2022.1035827

**Published:** 2022-11-08

**Authors:** Bao Gen Zhang, Xiao Fang Qian

**Affiliations:** ^1^School of Physical Education and Health, Zhao Qing University, Zhao Qing, China; ^2^Department of Physical Education, School of Humanities, Zhao Qing Medical College, Zhao Qing, China

**Keywords:** student engagement, obesity, weight self-stigma, self-determination theory, physical education

## Abstract

**Background:**

This is a cross-sectional in design. It involves the mediating effects of basic psychological need satisfaction in relation to the moderating effects of teacher autonomy support regarding weight self-stigma's effect on engagement among obese students in physical education classes.

**Methods:**

This study includes 165 Chinese high school obese students [mean age, 16.84 (±0.147) years], comprising 93 males (56.63%) and 72 females (43.63%), with a mean body mass index (BMI) of 30.453 (SD = 2.426). Participants completed the weight self-stigma questionnaire, basic psychological need satisfaction questionnaire, teacher autonomy support questionnaire, and student engagement questionnaire.

**Results:**

Weight self-stigma and engagement among obese students were mediated by basic psychological need satisfaction. Moreover, the mediated effect of basic psychological need satisfaction was moderated by teacher autonomy support.

**Conclusion:**

Weight self-stigma and basic psychological need satisfaction are the antecedents influencing the engagement of obese students. Notably, weight self-stigma not only directly blocks the engagement of obese students but also their engagement by hindering the acquisition of basic psychological need satisfaction. Teacher autonomy support can significantly reduce the negative impact of weight self-stigma on basic psychological need satisfaction and significantly promote engagement. Therefore, by promoting their physical education engagement, physical education teachers should strengthen the application of their supportive autonomous teaching strategies to help obese students meet their basic psychological needs.

## Introduction

In China, obesity rates have steadily increased in recent decades and are now a significant public health problem (Wang et al., 2007). Obesity has a tremendous impact on the health of adults, children, and adolescents (Jakab et al., 2020). It has many serious consequences, including some types of cancer (Altová, 2022), eating disorders (Bristow et al., 2022), cardiovascular diseases, type 2 diabetes (Stevens et al., 2012), thyroid dysfunction (Mahdavi et al., 2021), high blood pressure (Pileggi et al., 2021), depression, and anxiety (Werner-Seidler et al., 2017). Therefore, to reduce the negative effects of obesity, a series of interventions were made: exercise (Lee, 2021) through lifestyle, environmental, behavioral, pharmacologic, or surgical interventions (Aceves-Martins et al., 2022). Furthermore, moderate physical activity is one of the best tools to alleviate the complications associated with obesity (Apovian et al., 2015). China's education system relies almost entirely on test scores to evaluate its progress, which leads to more reading, examinations, and/or homework for Chinese children and less time for physical activities (Yu et al., 2012). Thus, physical education classes can be an ideal context to encourage the acquisition of healthy lifestyles throughout a student's life development (Langford et al., 2015). As a matter of fact, it has become the primary method by which students engage in physical exercise. Despite this, some studies show that the participation of obese students in physical education is not high, leading to a lack of physical activity during class.

Student engagement is a three-dimensional concept involving behavioral, emotional, and cognitive engagement (Fredricks et al., 2004). Behavioral engagement includes effort, exertion, and persistence, as well as activities such as answering the teacher's questions, actively engaging in various exercises, and carefully listening to the teacher (Skinner et al., 2008). Emotional engagement involves students' relationship with their teachers and peers, whether they view physical education as enjoyable or like it (Asogwa et al., 2020). Finally, cognitive engagement refers to an investment in learning that requires motivation, strategic learning skills, and problem-solving abilities (Fredricks et al., 2004). Student engagement has emerged as an essential construct in predicting self-esteem, happiness, and performance (Skinner et al., 2008). As such, student engagement is an essential prerequisite for students to engage in physical activities. In particular, without student engagement, there is no physical activity level; thus, the effect of physical and mental health promotion in physical education teaching is difficult to achieve without a physical activity level. However, obese students are less likely to flourish and be academically engaged than their healthier peers with a normal weight (Mccoy and Rupp, 2021). Research shows that low engagement among obese students is strongly associated with weight self-stigma. Specifically, weight stigma can be defined as the experience of verbal or physical abuse resulting from being overweight or obese (Wu and Berry, 2018). Obese students are stigmatized by their teachers, peers, and even family members, which can have detrimental physiological and psychological consequences (Stojadinovic et al., 2018). They are usually thought of as unmotivated, physically unattractive, stupid, or lacking willpower and discipline with regard to their body weight (Li and Rukavina, 2009). Studies show that obese students who are teased during physical activities prefer isolated, sedentary activities (Hayden-Wade et al., 2005), enjoy sports less, and engage in less physical activity than their peers (Storch et al., 2007). Unfortunately, obese students tend to internalize these stigmas and biases (Durso and Latner, 2008; Lillis et al., 2010; Alberga et al., 2016). Subsequently, this internalization of weight stigma causes children and adolescents to self-stigmatize themselves, exhibit negative emotional reactions, and discriminate against themselves (Corrigan et al., 2006), thereby increasing their risk of being socially undervalued and/or rejected (Puhl and Heuer, 2009). In fact, those who suffer from weight self-stigma have negative beliefs about themselves, experience negative feelings, and isolate themselves as a result (Hilbert et al., 2015). If other students withdraw their social support, exclude them, and discriminate against obese students, these behaviors negatively affect their physical and mental health and hinder study engagement. They will lead to frustration, demotivation, and further withdrawal from athletic activity, and the joy of movement will be lost (Pont et al., 2017).

The essential features of the physical education teaching process are interpersonal interactions. However, few studies explore the relationship between self-stigma and engagement among obese students in physical education classes, especially those involving obese Chinese students. Accordingly, based on the above research, this study's first hypothesis (Hypothesis 1) is put forward: Weight stigma negatively affects engagement among obese students in physical education classes.

A study found a positive association between weight stigma and less engagement in current exercise behavior (Vartanian and Shaprow, 2008). At the same time, stigma also entails psychosocial effects such as social isolation and feelings of rejection (Jung and Luck-Sikorski, 2019). Several studies have examined the relationship between weight stigma and engagement through the lens of self-determination theory (SDT). Notably, SDT is a motivation theory about human social situations that emphasizes three fundamental needs that must be fulfilled: autonomy, competence, and relatedness needs for optimal motivational functioning to occur along with subsequent positive outcomes (Ryan and Deci, 2000). Autonomy can be defined as one's need to experience a sense of willingness in one's actions, without pressure from other people, which can be expressed as one's need to feel like the originator and not the pawn of their behaviors; competence refers to an individual's perception of their own need to experience effectiveness in their interactions to accomplish certain tasks, and relatedness is defined as the extent to which one is connected and accepted by significant others (Rm Ryan, 2017). A social situation has the dual effect of meeting basic psychological needs. SDT argues that the more students' basic psychological needs are met, the more autonomous they become (Maldonado et al., 2019). In contrast, when individuals' basic psychological needs are not met (or partially met), they feel controlled and have more extrinsic motivation. As such, autonomous motivation is associated with more desirable engagement (Taylor et al., 2010), but extrinsic motivation is associated with higher frustration levels with autonomy, competence, and relatedness needs (Haerens et al., 2015; Jang et al., 2016). These findings are consistent with SDT's basic hypothesis, which postulates that basic psychological needs are intermediary during social situations affecting individual behavior. Thus, it is safe to say that stigmatization is a negative social situation that is not conducive to meeting basic psychological needs and consequently hinders individual engagement. A study with bariatric patients revealed that participants who experienced stigmatization experienced a thwarting of their basic psychological needs for autonomy, competence, and relatedness (Megías et al., 2018). This is associated with a lack of commitment, quitting activity, and adopting negative behaviors (Standage et al., 2005). Based on the above, this study's second hypothesis (Hypothesis 2) is presented: Weight self-stigma affects engagement among obese students in physical education classes through the mediating effect of basic psychological needs.

The goal of the physical education teaching process is to get students to improve their interpersonal interactions. These interpersonal interactions mostly include teachers and students. At the same time, the obese student's perceived stigma mainly comes from teachers and peers. Relevant research shows that teachers' supportive teaching strategies not only reduce the perception of stigma among obese students but also compensate for the basic psychological needs and frustrations caused by the lack of peer support (Wentzel and Asher, 1995). In physical education classes, teacher autonomy support is one of the aspects of teachers' supportive teaching strategies, which includes nurturing their inner motivational resources by respecting students' attitudes and suggestions, recognizing students' feelings, providing students with opportunities for choice, displaying patience to allow students the time they need for self-paced learning to occur (Reeve, 2009). Some studies based on SDT have demonstrated that teacher autonomy support can meet students' basic psychological needs (Fin et al., 2019; Leyton-Román et al., 2020), which is associated with a higher level of self-determined motivation and engagement in physical education classes (Valero-Valenzuela et al., 2021). Therefore, our third hypothesis (Hypothesis 3) is proposed: The association between weight self-stigma and basic psychological needs is moderated by the level of perceived teacher autonomy support.

**Figure 1:**
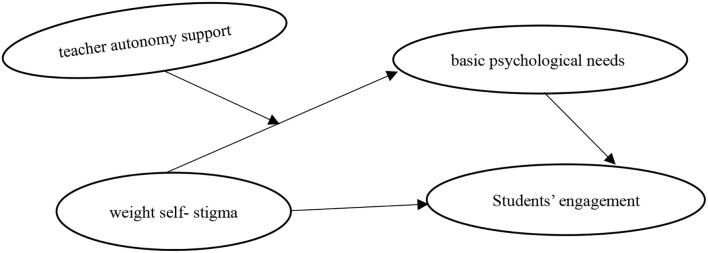
The hypothesized model.

Broadly, to investigate the relationship between weight self-stigma and engagement among obese students in a physical education class, we built a moderated mediation model (Figure 1) and verified the following three hypotheses: (1) weight self-stigma negatively affects engagement among obese students in physical education class; (2) weight self-stigma affects engagement among obese students in a physical education class through the mediating effect of basic psychological needs; and (3) the association between weight self-stigma and basic psychological needs is moderated by the level of perceived teacher autonomy support.

## Materials and methods

### Participants and procedures

Eight administrative districts in Shanghai were randomly selected as the survey areas. Six high schools were randomly selected from each administrative region, and a total of 48 schools were investigated. Each investigated school randomly selected a class called Senior One, Senior Two, and Senior Three. A total of 144 classes were surveyed, from which 2,583 students completed the questionnaire online. After screening invalid questionnaires for reasons such as missing data and non-conforming responses, a total of 2,217 valid questionnaires were finally retained. Among those 2,217 valid questionnaires, 165 students were defined as obese. Because their BMI was >28 (China, 2004), these 165 particularly obese students eventually became the research target of this study. Shanghai is an economically developed metropolis. All those obese students came from the city.

Table 1 presents the data of 165 obese students, comprising 93 males (56.63%) and 72 females (43.63%). Of them, 61 (36.96%), 56 (33.94%), and 48 (29.09%) were seniors, one, two, and three students, respectively, with a mean BMI of 30.453 (SD = 2.426), ranging from 28 to 39.12.

**Table 1:** Basic information on obese students (*n* = 165).

	**Senior one**		**Senior two**		**Senior three**		**Males**		**Females**	
	***n***	***N* (%)**	***N***	***N* (%)**	***n***	***N* (%)**	***n***	***N* (%)**	***n***	***N* (%)**
	61	36.96%	56	33.94%	48	29.09%	93	56.36%	72	43.63%
Age	15.14 ± 0.110		16.22 ± 0.175		17.19 ± 0.124		15.87 ± 0.776		15.91 ± 0.792	
BMI	29.221 ± 2.216		30.137 ± 2.394		30.341 ± 2.361		29.629 ± 1.350		31.199 ± 2.618	

Ethical approval was obtained from the Ethics Committee of Zhao Qing University. This approval was administered through each district's education department, which then contacted each school. We asked for permission *via* informed consent, which the headmaster of each school signed before we collected the data. In addition, we also obtained signed informed consent from the student's parents. Subsequently, we trained the principals of physical education teaching at each school, who were then responsible for the questionnaire survey of their school. The training was intended to explain to the students that the questionnaire survey would only be used for the paper research work and that there were no right or wrong answers. Finally, the students were required to answer truthfully, and they had the right to choose whether to answer or not.

### Measures

#### Weight stigma questionnaire

This study used the Chinese version of the Weight Self-Stigma Questionnaire (C-WSSQ) to assess stigma (Lin and Lee, 2017). The questionnaire included two dimensions in its assessment and comprised 12 items, which were scored on a five-point Likert scale, ranging from 1 (strongly disagree) to 5 (strongly agree). Example items from the 12-item scale included “I feel guilty about my obesity” and “Because of my obesity, others think I lack self-control.” In the present study, Cronbach's alpha was 0.927, and the split-half coefficient was 0.933.

#### Basic psychological need satisfaction in the physical education class

Psychological need satisfaction was measured using the Chinese version of the basic psychological needs in the physical education scale (Zhang Bao Gen, 2020). This 12-item scale assessed three dimensions: autonomy satisfaction (“I feel that the way classes are taught is a true expression of who I am”), competence satisfaction (“I feel that I improve even in the tasks considered difficult by most of the children”), and relatedness satisfaction (“My relationships with my classmates are very friendly”). Participants responded to items from 1 (strongly disagree) to 5 (strongly agree). In the present study, Cronbach's alpha was 0.936, and the split-half coefficient was 0.919.

#### Teacher autonomy and support in the physical education class

A teacher autonomy support scale was used to measure teacher autonomy support (Reeve et al., 2003). Example items from the 7-item scale include “My PE teacher allows me to choose between different exercises” and “My PE teacher answers me when I express my opinion.” In the present study, the autonomy teacher support scale's Cronbach's alpha was 0.928, and the split-half coefficient was 0.925.

#### Student engagement

Student engagement was measured using the student engagement scale in physical education (Agbuga, 2015), which had been validated among Chinese students (Zhang Bao Gen, 2020). This 13-item scale assessed three dimensions: behavioral engagement (“I try hard to do well in class.”), emotional engagement (“When I am in class, I feel good”), and cognitive engagement (“I ask myself questions while practicing to monitor my performance”). Participants responded to items from 1 (strongly disagree) to 5 (strongly agree). In the present study, Cronbach's alpha was 0.917, and the split-half coefficient was 0.921.

A confirmatory factor analysis was conducted to test the validity of the whole set of measurement tools, which is composed of four sub-measurement tools (Table 2). The scale's structural validity was adequate [x^2^/df = 4.142, goodness-of-fit index (GFI) = 0.943, normed fit index (NFI) = 0.939, incremental fit index (IFI) = 0.948, comparative fit index (CFI) = 0.951, root mean square error of approximation (RMSEA) = 0.063, and standardized root mean square residual (SRMR) = 0.047]. The scale's structural validity was adequate (Hu and Bentler, 1999).

**Table 2:** Confirmatory factor analysis of the whole set of measurement tools.

**Variable**	**Confirmatory factor analysis**							
	***x^2^/df***	**GFI**	**NFI**	**IFI**	**RFI**	**CFI**	**RMSEA**	**SRMR**
Measurement tool	4.142	0.943	0.939	0.939	0.948	0.952	0.063	0.047

### Statistical analyses

Regarding statistical software, SPSS software (version 25.0) was used for correlations and descriptive statistics. The PROCESS for SPSS was used for mediation analyses, and the structural equation model was used to test the fit of the mediation path model.

### Control and inspection of common method variance

Notably, only self-reported data were collected, which could have a common bias (Podsakoff et al., 2003). In order to reduce common bias, some necessary controls were carried out, such as using reverse expressions for some items. Harman's single-factor analysis was used to include weight self-stigma, basic psychological needs, teacher autonomy support, and student engagement items in the exploratory factor analysis. In the principal component analysis without a varimax rotation, 15 factors demonstrated eigenvalues >1, and the first factor explained 27.133% of the variance, with < 40% critical value. These figures indicate no significant common method bias. Therefore, the common method of deviation in this study was acceptable.

## Results

### Descriptive statistical and correlation analysis

A Pearson's product-moment correlation analysis was used to analyze weight self-stigma, basic psychological needs, teacher autonomy support, and student engagement. The results showed Table 3 weight self-stigma was significantly negatively correlated with basic psychological needs, teacher autonomy support, and student engagement (*r* = −0.237, *p* < 0.01; *r* = −0.220, *p* < 0.01; *r* = −0.327, *p* < 0.01). Basic psychological needs were significantly positively correlated with teacher autonomy support and student engagement (*r* = 0.614, *p* < 0.01; *r* = 0.675, *p* < 0.01). Moreover, teacher autonomy support was significantly positively correlated with student engagement (*r* = 0.541, *p* < 0.01).

**Table 3:** Mean, standard deviation, and correlation coefficient of each variable.

	**M ±SD**	**Range**	**1**	**2**	**3**	**4**
1. Weight self-stigma	2.790 ± 0.882	1–5	1			
2. Basic psychological needs	4.338 ± 0.646	1–5	−0.237**	1		
3. Teacher autonomy support	4.355 ± 0.682	1–5	−0.220**	0.614**	1	
4. Student engagement	4.483 ± 0.635	1–5	−0.327**	0.675**	0.541**	1

^**^Significant correlation at the 0.01 level (Two-tailed test), p < 0.01.

### The test of mediating effect

Table 4 presents the results for Hypotheses 1 and 2. Regression analysis showed that self-stigma negatively affected engagement among obese students (M1), supporting Hypothesis 1. The regression results also revealed that weight self-stigma negatively affected basic psychological need satisfaction (M3). The regression results from M2 demonstrated that basic psychological need satisfaction significantly affected engagement among obese students. Simultaneously, weight self-stigma still negatively affected engagement among obese students. Therefore, we can conclude that basic psychological need satisfaction had a partial mediating effect, and this effect was significant, supporting Hypothesis 2. The structural mode model was used to test the model fit of the intermediary path of weight self-stigma → basic psychological need satisfaction → student engagement. Specifically, this model showed an ideal degree of fit (*x*^2^*/df* = 4.57, GFI = 0.949, NFI = 0.937, IFI = 0.933, RFI = 0.916, CFI = 0.941, TLI = 0.956, RMSEA = 0.043, and SRMR = 0.037) (Hu and Bentler, 1999).

**Table 4:** A hierarchical regression analysis of the mediating effect of basic psychological needs.

**Predictor**	**Student engagement**		**Basic psychological need satisfaction**		
	**M1**	**M2**	**M3**	**M4**	**M5**
Weight self-stigma	−0.327**	−0.177**	−0.237**	−0.107**	−0.630**
Basic psychological need satisfaction		0.633**			
Teacher autonomy support				0.591**	0.421**
Basic psychological need satisfaction *Teacher autonomy support					0.519**
*R^2^*	0.107	0.485	0.056	0.385	0.395
*F*	45.920	179.970	22.816	121.214	82.775

^**^Significant correlation at the 0.01 level (Two-tailed test), p < 0.01.

### Moderated mediation effect test

**Figure 2:**
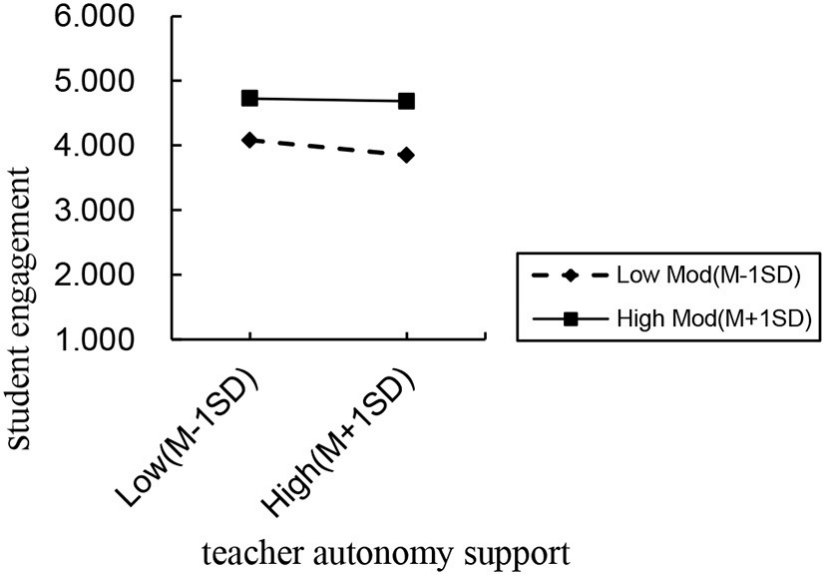
Simple slope analysis.

The regression results of M4 and M5 demonstrate that teacher autonomy support significantly positively affects basic psychological need satisfaction. Moreover, the interaction between weight self-stigma and teacher autonomy significantly positively affects basic psychological need satisfaction. A simple slope analysis revealed that (Table 5 and Figure 2) with a higher level of perceived teacher autonomy support (M+1 SD) among obese students, weight self-stigma could not significantly negatively affect basic psychological need satisfaction. With low perceived teacher autonomy support (M−1 SD), weight self-stigma significantly negatively affected their basic psychological needs. The above information reveals that a higher level of teacher autonomy support can buffer the negative effect of weight self-stigma on basic psychological need satisfaction among obese students. Therefore, the association between weight self-stigma and basic psychological needs was moderated by the level of perceived teacher autonomy support, and thus, Hypothesis 3 was supported.

**Table 5:** Moderating effect of different levels of teacher autonomy support on the relationship between weight self-stigma and engagement among obese student.

**Teacher autonomy support**	**Intermediate affect value**	**SE**	**LLCI**	**ULCI**
M – SD	−0.084	0.038	−0.161	−0.013
M	−0.049	0.021	−0.094	−0.011
M + SD	−0.016	0.023	−0.067	0.025

## Discussion

### Weight Self-stigma directly and significantly negatively affects engagement

In this study, we found that weight self-stigma negatively affects engagement directly among obese students, which is in line with previous studies (Myre et al., 2021). Weight self-stigma is a type of negative body image and may lead to obese students feeling incompetent and powerless (Tomiyama et al., 2018). A previous study has shown that weight self-stigma was associated with lower self-esteem (Alahmari et al., 2019) and poor self-esteem (Carr and Friedman, 2005; Tomiyama et al., 2018). Notably, weight self-stigma is a particularly negative emotion, and it is an accumulation of negative emotions over time that ultimately leads to depression. Unpleasant experiences cause more psychological problems and create a vicious circle, which some researchers have associated with lower engagement (Mouchacca et al., 2013; Meadows and Bombak, 2019; Sabiston et al., 2019). The above analysis shows that weight self-stigma significantly affects engagement among obese students in physical education classes.

### Intermediary role of basic psychological need satisfaction

This study has found that basic psychological need satisfaction is an intermediary between weight self-stigma and engagement among obese students. It also shows that basic psychological need satisfaction is the key factor in understanding the relationship between weight self-stigma and engagement among obese students in physical education classes. Weight self-stigma is averse to the satisfaction of basic psychological needs, reducing engagement among obese students. This finding is consistent with the basic hypothesis of SDT (Rm Ryan, 2017), which states that people are more receptive to changing their behavior when their basic psychological needs are met; otherwise, it impedes behavioral engagement. In turn, neglecting one's basic psychological needs and frustration are factors that lead people to stop participating in physical activity (Megías et al., 2018). This may be because weight self-stigma can be viewed as a study stressor. The generalized tension theory holds that when individuals experience stress and tension, they produce negative emotions such as anxiety, depression, and fear (Agnew and White, 1992); these negative emotions frustrate basic psychological needs. In addition, people struggling with self-stigma are ostracized by society, which harms their sense of belonging. Not feeling included is one of the main factors contributing to the failure to meet students' basic psychological needs. According to the theory of interpersonal perception, when an individual is excluded and marginalized by the outside world, they feel lonely due to emotional damage, which further causes them to withdraw from social activities (Schutz, 1958). If an individual feels lonely in interpersonal communication, it causes depression and social withdrawal and affects their subsequent behaviors.

### Moderating effects of teacher autonomy support

The results show that teacher autonomy moderates the relationship between weight self-stigma and engagement among obese students. Those who report receiving more emotional support from teachers are more likely to be able to meet their most basic psychological requirements while dealing with weight self-stigma. This may be because a higher level of perceived teacher autonomy support among obese students in physical education classes makes them believe they still have room for choice and change. In this case, they can experience more positive emotions and remain optimistic. According to the expansion construction theory, positive emotions can help individuals expand their thinking and construct psychological resources to deal with the outside world (Jie, 2010).

Furthermore, positive emotions and optimism may have more self-determined motivations (Bartholomew et al., 2011). According to the SDT, more self-determined motivations lead to adaptive consequences. Therefore, teachers' autonomy support can help obese students maintain more self-efficacy in the face of perceived stigma and enhance their adaptability for engagement. This shows that teacher autonomy support can reduce the negative influence of self-stigma on basic psychological need satisfaction and further promote engagement among obese students in physical education classes.

### Theoretical contributions and practical significance

This study discusses the relationship between weight self-stigma and engagement among obese students in physical education classes. It also features the construction of a moderated mediation model and reveals the internal mechanism of weight self-stigma on engagement in physical education classes. We found that basic psychological need satisfaction mediates how weight self-stigma affects engagement among obese students, which has not been reported in previous studies. Our results are consistent with the SDT postulates, which help raise awareness among physical education teachers regarding their potential responsibility in the struggle against weight stigma in their classes. Finally, this study has significant theoretical value for understanding the causes of engagement among obese students in high school, which enriches the theoretical research on and application of the effects of SDT.

In practice, this study's findings highlight that autonomy support is an essential skill for physical education teachers to promote engagement among their obese students. We then suggest that physical education teachers utilize the following autonomy-supportive skills, including providing choices to students with obesity, offering a rationale for study tasks, giving them opportunities to take the initiative, providing non-controlling competence feedback, and acknowledging their feelings and perspectives (Mageau and Vallerand, 2003). Physical education teachers should try their best to make these students more socially integrated into their peer group, strengthening their ties. This measure should make it difficult for potential peer bullies to obtain opportunities to reinforce their stigma and help teachers and students recognize obese students. Lastly, it is vital to promote legislation that prohibits weight-based discrimination.

### Limitations and prospects for future research

Despite all the strengths, the present study did have some limitations. First, we only considered the mediating effect of basic psychological need satisfaction, but there may be other mediating variables, such as body image, self-esteem, loneliness, distress, anxiety, and depression, among others, which need to be further explored. Second, other moderate variables, such as the teacher's structural support, teacher's relatedness support, and peer support, also need to be further explored. Third, there were methodological limitations, including self-reports for data collection, the cross-sectional nature of the work, and the fact that we tested a single model in just one sample. Therefore, we suggest future research studies with similar models with experimental and/or qualitative methodologies. Fourth, obese students are more likely to perceive a controlling teaching style than a supportive one. Given this, future research should focus on exploring effective teaching strategies to reduce the controlling style of teaching. Finally, and most importantly, engagement among obese students in physical education classes should be improved, and future research should investigate how survey results correlate to actual teaching practice.

## Conclusion

Using SDT, the novel findings from the present study showed that weight self-stigma not only directly affects engagement among obese students but also impacts the mediating effect of basic psychological need satisfaction on engagement. Moreover, perceived teacher autonomy support can moderate the effect of weight self-stigma on their engagement. In practice, our results showed that physical education teachers should create an environment supporting autonomy that meets obese students' three basic psychological needs.

## Data availability statement

The original contributions presented in the study are included in the article/supplementary material, further inquiries can be directed to the corresponding author/s.

## Ethics statement

Ethical approval was obtained from the Ethics Committee of Zhao Qing University. Participant's parents provided written informed consent.

## Author contributions

BZ and XQ had substantial contributions to the conception and design of the work. BZ drafting the work and revising it critically for important intellectual content. The manuscript was written through the contributions of all authors. All authors have given approval to the final version of the manuscript.

## Conflict of interest

The authors declare that the research was conducted in the absence of any commercial or financial relationships that could be construed as a potential conflict of interest.

## Publisher's note

All claims expressed in this article are solely those of the authors and do not necessarily represent those of their affiliated organizations, or those of the publisher, the editors and the reviewers. Any product that may be evaluated in this article, or claim that may be made by its manufacturer, is not guaranteed or endorsed by the publisher.
